# Regulation of m^6^Am RNA modification and its implications in human diseases

**DOI:** 10.1093/jmcb/mjae012

**Published:** 2024-03-20

**Authors:** Hao Jin, Zhouyuanjing Shi, Tianhua Zhou, Shanshan Xie

**Affiliations:** Children's Hospital, Zhejiang University School of Medicine, National Clinical Research Center for Child Health, Hangzhou 310052, China; Department of Cell Biology, Zhejiang University School of Medicine, Hangzhou 310058, China; Department of Gastroenterology, The Second Affiliated Hospital, Zhejiang University School of Medicine, Hangzhou 310020, China; Department of Cell Biology, Zhejiang University School of Medicine, Hangzhou 310058, China; Department of Gastroenterology, The Second Affiliated Hospital, Zhejiang University School of Medicine, Hangzhou 310020, China; Center for Medical Research and Innovation in Digestive System Tumors, Ministry of Education, Hangzhou 310020, China; Children's Hospital, Zhejiang University School of Medicine, National Clinical Research Center for Child Health, Hangzhou 310052, China

**Keywords:** m^6^Am RNA modification, gene regulation, obesity, viral infection, cancer

## Abstract

*N* ^6^,2′-*O*-dimethyladenosine (m^6^Am) is a prevalent modification frequently found at the 5′ cap-adjacent adenosine of messenger RNAs (mRNAs) and small nuclear RNAs (snRNAs) and the internal adenosine of snRNAs. This dynamic and reversible modification is under the regulation of methyltransferases phosphorylated CTD interacting factor 1 and methyltransferase-like protein 4, along with the demethylase fat mass and obesity-associated protein. m^6^Am RNA modification plays a crucial role in the regulation of pre-mRNA splicing, mRNA stability, and translation, thereby influencing gene expression. In recent years, there has been growing interest in exploring the functions of m^6^Am and its relevance to human diseases. In this review, we provide a comprehensive overview of the current knowledge concerning m^6^Am, with a focus on m^6^Am-modifying enzymes, sequencing approaches for its detection, and its impacts on pre-mRNA splicing, mRNA stability, and translation regulation. Furthermore, we highlight the roles of m^6^Am in the context of obesity, viral infections, and cancers, unravelling its underlying regulatory mechanisms.

## Introduction

*N* ^6^,2′-*O*-dimethyladenosine (m^6^Am) is a prevalent and abundant post-transcriptional RNA modification detected at the transcriptional start site (TSS) of eukaryotic messenger RNAs (mRNAs), a subset of small nuclear RNAs (snRNAs), and viral RNAs (Wei et al., 1975; Boulias and Greer, 2023). It is also found at the internal adenosine of U2 snRNA (Boulias and Greer, 2023). Compared with the well-studied *N*^6^-methyladenosine (m^6^A), m^6^Am shows differences in many aspects, including structure and abundance (Table 1). Since its discovery in 1975, m^6^Am has been found to account for ∼30% of cellular mRNAs and even more prevalent in certain viral RNAs (Wei et al., 1975). Across human tissues, the ratio of m^6^Am to adenosine in total RNA ranges from 0.0036% to 0.0169%, whereas in mouse tissues, this ratio ranges from ∼0.02% to 0.07% (Liu et al., 2020). This variation underscores the diverse levels of m^6^Am across species, which is also reflected by the fact that m^6^Am-modified genes in the same tissue vary greatly in different species (Liu et al., 2020).

**Table 1:** The characteristics of m^6^Am and m^6^A RNA modifications.

Characteristics	m^6^Am	m^6^A
Modification position	2′ and *N*^6^ positions	*N* ^6^ position
RNA species	mRNA and snRNA	mRNA, lncRNA, snRNA, rRNA, and tRNA
Abundance in mRNA	0.015%–0.06% (m^6^Am/A)	0.15%–0.6% (m^6^A/A)
Location at mRNA	TSS	Near stop codon
Sequence motif	Genomic BCA motif	DRACH motif
Methyltransferases	PCIF1 and METTL4	METTL3, METTL5, METTL16, and ZCCHC4
Demethylases	FTO	FTO and ALKBH5

A, adenosine; ZCCHC4, zinc finger CCHC-type containing 4; ALKBH5, AlkB homologue 5.

**Figure 1:**
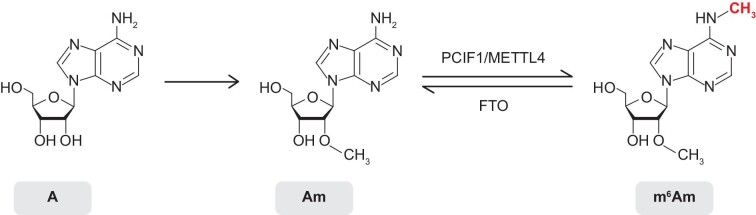
Reversible regulation of m^6^Am RNA modification. m^6^Am is formed by the methyltransferase PCIF1 or METTL4 on the basis of Am and is removed by the demethylase FTO. A, adenosine.

The dynamic and reversible characteristics of m^6^Am RNA modification are governed by specific methyltransferases and demethylases (Figure 1; Sendinc and Shi, 2023). Phosphorylated CTD interacting factor 1 (PCIF1), also known as cap-specific adenosine methyltransferase, is responsible for converting 2′-*O*-methyladenosine (Am) to m^6^Am in an *S*-adenosylmethionine-dependent manner (Akichika et al., 2019; Boulias et al., 2019; Sendinc et al., 2019; Sun et al., 2019). Another m^6^Am methyltransferase is methyltransferase-like protein 4 (METTL4), which mediates internal m^6^Am modification in U2 snRNA (Chen et al., 2020; Goh et al., 2020; Luo et al., 2022). The removal of m^6^Am RNA modification is promoted by the demethylase fat mass and obesity-associated protein (FTO), which is capable of removing m^6^Am from both mRNAs and snRNAs (Mauer et al., 2017; Wei et al., 2018, 2019).

Advances in next-generation sequencing-based approaches have provided insights into the global m^6^Am methylome and its dynamics under stress and pathological conditions (Koh et al., 2019; Linder et al., 2015; Sendinc et al., 2019; Sun et al., 2021). These techniques not only offer valuable information on the distribution of m^6^Am across the transcriptome but also shed light on its biological functions and clinical implications. Notably, m^6^Am has been found to be involved in modulating pre-mRNA splicing (Chen et al., 2020; Goh et al., 2020), mRNA stability (Mauer et al., 2017; Boulias et al., 2019), and translation (Sendinc et al., 2019; Sikorski et al., 2020; Ben-Haim et al., 2021; Zhuo et al., 2022), thereby regulating gene expression.

In this review, we summarize the current state of research on m^6^Am-modifying enzymes, sequencing methodologies for m^6^Am detection, and the effects of m^6^Am on pre-mRNA splicing, mRNA stability, and translation regulation. Furthermore, we highlight the crucial roles and underlying regulatory mechanisms of m^6^Am in obesity, viral infections, and cancers. By exploring these aspects, we aim to contribute to a deeper understanding of the multifaceted functions of m^6^Am and its potential implications in human diseases.

## Reversible m^6^Am RNA modification by methyltransferases and demethylases

The abundance of m^6^Am modification on RNA varies among transcripts and is regulated by the activity of methyltransferases and demethylases (Sun et al., 2023). PCIF1 is an evolutionarily conserved cap-adjacent m^6^Am methyltransferase derived from prokaryotic M.EcoKI/M.TaqI (Iyer et al., 2016). It features a tryptophan-rich WW domain in the N-terminal region, facilitating interaction with the Ser5-phosphorylated C-terminal domain of RNA polymerase II, thus enabling PCIF1 to catalyze the addition of m^6^Am at the TSS of mRNAs during transcriptional elongation (Fan et al., 2003; Akichika et al., 2019). The methyltransferase domain in the C-terminus of PCIF1 contains a key NPPF motif for its catalytic activity (Akichika et al., 2019). *In vitro* methylation assays demonstrated that both adenosine and Am are catalytic substrates for PCIF1, undergoing methylation to form m^6^A and m^6^Am, respectively (Akichika et al., 2019; Boulias et al., 2019). However, the binding affinity of PCIF1 for cap-adjacent Am is ∼8-fold greater than that for cap-adjacent adenosine. Additionally, RNA mass spectrometry failed to detect adenosine- and m^6^A-initiated mRNAs *in vivo* (Akichika et al., 2019). Consequently, the current hypothesis posits that PCIF1 installs an additional methyl group at the *N*^6^ position of cap-adjacent Am, converting it into cap m^6^Am.

The MT-A70 family member METTL4, like its paralogues METTL3 and METTL14, is conserved from yeast to humans (Iyer et al., 2016; Chen et al., 2020). METTL4 is an internal m^6^Am methyltransferase that contains a DPPW motif required for methyltransferase activity (Goh et al., 2020). It mediates *N*^6^-methylation of A30 on U2 snRNA in the context of an AAG motif when this site is 2′-*O*-methylated both *in vivo* and *in vitro* (Karijolich and Yu, 2010; Chen et al., 2020; Goh et al., 2020; Luo et al., 2022). Notably, whether METTL4 has additional substrates besides U2 snRNA remains to be explored in the future. In addition, the crystal structure of *Arabidopsis* METTL4 presented a classic sandwich fold in the methyltransferase domain, indicating that METTL4 is capable of catalyzing methylation independently (Luo et al., 2022). In mammalian cells, *METTL4* depletion results in a significant reduction in internal m^6^Am but does not affect the level of cap m^6^Am, while knockout of *PCIF1* has no impact on the level of internal m^6^Am (Chen et al., 2020), indicating that METTL4 and PCIF1 are distinct m^6^Am methyltransferases that target different RNA species and regions.

FTO was initially identified as an RNA demethylase that targets m^6^A (Jia et al., 2011). It was recognized as an m^6^Am demethylase several years later (Mauer et al., 2017). It is a human homologue of *Escherichia coli* AlkB dioxygenase and relies on Fe^2+^ and alpha-ketoglutarate to remove m^6^Am from both mRNAs and snRNAs (Mauer et al., 2017; Wei et al., 2018, 2019). FTO-mediated demethylation of m^6^Am varies in different cellular components: nuclear-distributed FTO primarily targets m^6^Am in snRNAs, while cytoplasmic FTO predominantly acts on m^6^Am-modified mRNAs (Wei et al., 2018). Despite its function as an m^6^Am demethylase, FTO is better known for removing m^6^A modification from RNAs. *In vitro* studies revealed that the catalytic efficiency (*K*_cat_/*K*_m_) of FTO for m^6^Am is much greater than that for m^6^A in synthetic oligonucleotides (Mauer et al., 2017). However, when purified FTO was applied to cellular mRNAs, nearly equivalent absolute numbers of m^6^Am and m^6^A were demethylated (Zhang et al., 2019). Further exploration of substrate specificity and the molecular basis of FTO recognizing m^6^A and m^6^Am sites will provide valuable insights into the fine-tuned regulation of these RNA modifications and their impacts on cellular processes.

## Methods for m^6^Am mapping in the transcriptome

**Figure 2:**
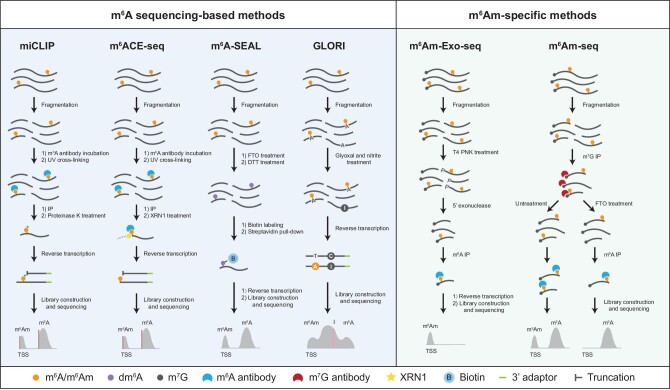
Current and potential methods available for m^6^Am profiling. Existing m^6^A sequencing-based methods, such as miCLIP and m^6^ACE-seq, can provide comprehensive profiling of m^6^Am in the transcriptome, while emerging techniques like m^6^A-SEAL and GLORI hold promise for future applications depending on the precise location of m^6^Am sites at the TSS. m^6^Am-specific methods, including m^6^Am-Exo-seq and m^6^Am-seq, have been developed for sequencing applications. dm^6^A, *N*^6^-dithiolsitolmethyladenosine; m^7^G, *N*^7^-methylguanosine; DTT, dithiothreitol; XRN1, 5′–3′ exoribonuclease 1; IP, immunoprecipitation; PNK, polynucleotide kinase.

For a long time, our understanding of transcripts harboring m^6^Am was limited due to the lack of high-throughput techniques. As a result, the specific biological functions of m^6^Am RNA modification on individual transcripts also remain unclear. A widely adopted approach for mapping m^6^Am involves the examination of modified nucleotides or peaks proximal to the TSS through m^6^A immunoprecipitation-dependent sequencing data, typically identifying m^6^Am locations (Cesaro et al., 2023). Several m^6^A sequencing techniques have been developed, including m^6^A individual–nucleotide–resolution cross-linking and immunoprecipitation (miCLIP), m^6^A-cross-linking–exonuclease-sequencing (m^6^ACE-seq), FTO-assisted m^6^A selective chemical labeling (m^6^A-SEAL), and glyoxal and nitrite-mediated deamination of unmethylated adenosines (GLORI) (Linder et al., 2015; Koh et al., 2019; Wang et al., 2020; Liu et al., 2023; Figure 2). Notably, miCLIP and m^6^ACE-seq have been utilized for profiling m^6^Am RNA modification, while other techniques also hold potential for identifying m^6^Am RNA modification. Additionally, m^6^Am–exonuclease sequencing (m^6^Am-Exo-seq) has been developed specifically for the sequencing of m^6^Am RNA modification. Building on m^6^A sequencing, this method enriches mRNA 5′ ends by pre-treating uncapped fragments with 5′ exonuclease (Sendinc et al., 2019). Through these approaches, thousands of modified sites have been annotated in the transcriptome. Furthermore, it has been observed that cap m^6^Am is commonly installed in a BCA (B = C, T, or G) consensus sequence within DNA (Linder et al., 2015; Koh et al., 2019).

Notably, these methods may not provide m^6^Am information with high confidence because they cannot distinguish cap m^6^Am from nearby m^6^A (Dominissini et al., 2012). While the application of *PCIF1* knockout was designed to address this concern, its effectiveness may be constrained by potentially incomplete elimination of m^6^Am (as PCIF1 might not be the sole methyltransferase for m^6^Am) and the interference of highly abundant m^6^A sites in close proximity, particularly in low-resolution techniques. To overcome this limitation, Sun et al. (2021) developed m^6^Am sequencing (m^6^Am-seq), which reduced the demethylase activity of FTO *in vitro* by omitting l-ascorbic acid and thus allowed FTO to selectively demethylate cap m^6^Am (>95%) without impacting internal m^6^A, enabling discrimination between m^6^Am and m^6^A near mRNA 5′ cap (Sun et al., 2021). m^6^Am-seq identified 1652 cap m^6^Am peaks from 1635 transcripts in HEK293T cells and has been used to profile the m^6^Am methylome under heat shock or hypoxia conditions, revealing hundreds of altered cap m^6^Am peaks (Sun et al., 2021). These data validate the hypothesis of m^6^Am dynamics under stress conditions and provide further insight into the role of m^6^Am in the pathogenesis of diseases.

## Biological consequences of m^6^Am on gene expression

Given the crucial role of RNA in the central dogma, m^6^Am RNA modification is naturally linked to the regulation of gene expression. At the molecular level, m^6^Am is considered a key regulator of mRNA metabolism, including pre-mRNA splicing, mRNA stability, and translation, although some reports showed different results.

### m^6^Am RNA modification in pre-mRNA splicing

U2 snRNA is an essential component of the major spliceosome, which is involved in branch point selection and catalysis during pre-mRNA splicing (Lee and Rio, 2015). Notably, m^6^Am modification on the A30 of U2 snRNA is positioned directly upstream of the branch point recognition sequence, suggesting a potential role for m^6^Am in the splicing activity of the spliceosome (Chen et al., 2020). In *METTL4* knockout cells, a substantial number of splicing events are significantly altered (Chen et al., 2020; Goh et al., 2020). Moreover, FTO inhibition facilitates the accumulation and assembly of m^6^Am-modified snRNAs into small nuclear ribonucleoproteins, leading to enhanced exon inclusion in the splicing process (Mauer et al., 2019). These data suggest that m^6^Am epitranscriptomic information in snRNAs regulates pre-mRNA splicing.

### m^6^Am RNA modification in mRNA stability

One of the most well-known functions of RNA methylation is to regulate RNA stability (Boo and Kim, 2020), and m^6^Am appears to play an important role in this process. For instance, PCIF1 has been shown to be involved in regulating several processes, including obesity, viral infection, and cancer progression, by catalyzing m^6^Am to stabilize substrate mRNAs (Pandey et al., 2020; Zhang et al., 2021; Wang et al., 2023a, b). Studies showed that mRNAs starting with m^6^Am have a half-life time ∼1.5-fold longer compared to those starting with Am, Cm, Gm, or Um (collectively referred to as Nm), contributing to the greater abundance of m^6^Am-modified transcripts in HEK293T cells (Mauer et al., 2017; Boulias et al., 2019). Synthetic mRNAs initiated with m^6^Am also exhibited enhanced stability compared to those initiated with Am in HEK293T cells (Mauer et al., 2017). When FTO was exogenously expressed with a nuclear export signal to specifically reduce m^6^Am levels, m^6^Am-initiated mRNAs exhibited a shorter half-life time compared to Nm-initiated mRNAs (Mauer et al., 2017). Furthermore, RNA-sequencing (RNA-seq) data revealed that *FTO* knockdown leads to a greater abundance of m^6^Am-initiated mRNAs compared to Nm-initiated mRNAs in HEK293T cells, suggesting that the increased level of m^6^Am facilitates mRNA stabilization (Mauer et al., 2017). The mechanism underlying the increased stability of m^6^Am-initiated mRNAs may be associated with resistance to the mRNA-decapping enzyme DCP2-mediated decapping and microRNA-mediated mRNA degradation (Mauer et al., 2017).

However, a previous study concluded that cap m^6^Am and m^6^A near the 5′ cap, collectively referred to as ‘methylated TSS’, were not associated with mRNA stability according to the evaluation of the mRNA half-life (Schwartz et al., 2014). Additionally, an individual case involving *EGFP* mRNA with cap m^6^Am or Am transfected into HeLa cells did not show any significant difference between m^6^Am-initiated and Am-initiated mRNAs in their abundance (Zhuo et al., 2022). Similarly, two other independent reports showed that reduced m^6^Am abundance caused by *PCIF1* deletion did not affect the abundance of m^6^Am-modified mRNAs in HEK293T and MEL624 melanoma cells (Akichika et al., 2019; Sendinc et al., 2019). An explanation for these seemingly conflicting results could be that *PCIF1* knockout predominantly facilitates the stability of mRNAs with low expression levels while exerting minimal effects on highly expressed mRNAs (Boulias et al., 2019).

### m^6^Am RNA modification in mRNA translation

The fact that m^6^Am is located proximal to the 5′ cap of mRNAs suggests its potential role in affecting mRNA translation. A prior investigation suggested that the presence of ‘methylated TSS’ might augment the translation efficiency of modified transcripts (Schwartz et al., 2014). Moreover, comprehensive proteomic profiling analyses demonstrated that cap m^6^Am RNA modification enhances the protein expression of modified mRNAs in mouse embryonic stem cells (Ben-Haim et al., 2021). In various cell lines, including 3T3-L1, HeLa, and JAWS II, *in vitro*-transcribed *Gaussia* luciferase mRNA starting with m^6^Am had higher protein expression levels, indicating that m^6^Am may improve the efficiency of mRNA translation (Sikorski et al., 2020).

However, *in vivo* expression of *EGFP* mRNA in MEL624 and HeLa cells, as well as *in vitro* translation assays of firefly luciferase, showed that cap m^6^Am might suppress translation efficiency (Sendinc et al., 2019; Zhuo et al., 2022). Proteomic analysis revealed that proteins encoded by m^6^Am-modified mRNAs were upregulated after *PCIF1* deletion in MEL624 cells (Sendinc et al., 2019). Silencing *PCIF1* also enhanced the translation of its target mRNAs in BGC-823 gastric cancer cells (Zhuo et al., 2022). Additional studies employed ribosome profiling and RNA-seq to assess translation efficiency in control and *PCIF1* knockout HEK293T cells, but no substantial changes in the translation of m^6^Am-initiated mRNAs were observed following *PCIF1* depletion (Akichika et al., 2019; Boulias et al., 2019).

The conflicting roles of m^6^Am RNA modification in mRNA stability and translation likely result from a combination of factors, including variations in cell types, mRNA targets, experimental design, and analytical methods. Certain experimental approaches possess inherent limitations. For instance, the use of synthetic RNAs to study the effects of m^6^Am on RNA metabolism might not fully reflect what happens *in vivo*. Genetic modifications, such as *PCIF1* knockout, may yield distinct outcomes in different cellular environments, and long-term knockout may cause potentially redundant effects. Additionally, the intricate interplay between m^6^Am and other DNA/RNA modifications and the context-dependent characteristics of regulatory mechanisms could further contribute to conflicting findings. These could be addressed through employment of diverse cell types, standardized experimental conditions, and complementary techniques. Collaborative efforts and meta-analyses could also serve as valuable strategies to reconcile conflicting results across studies.

## The roles of m^6^Am RNA modification in diseases

**Figure 3:**
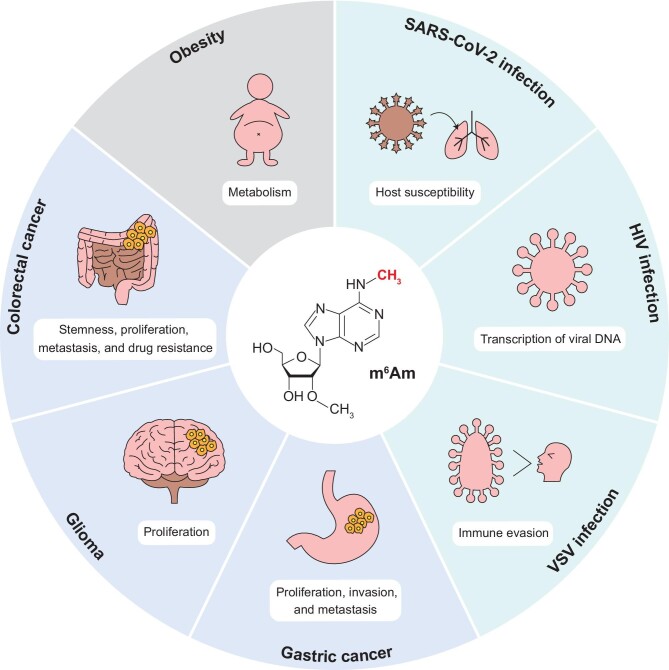
Diseases related to dysregulation of m^6^Am RNA modification and its modifying enzymes.

Post-transcriptional regulation plays a key role in the onset and development of various diseases, and RNA modifications and their associated enzymes are critical players in the pathogenesis of diseases. Dysregulation of m^6^Am and its modifying enzymes has been observed in a wide range of human diseases (Figure 3).

### m^6^Am RNA modification and obesity

Obesity has a profound impact on human health, increasing susceptibility to various diseases (Blüher, 2019). A study utilizing *PCIF1* knockout mice revealed a substantial decrease in m^6^Am modification in mRNAs, concomitant with a reduction in body weight (Pandey et al., 2020). This finding implies a potential link between PCIF1-mediated m^6^Am RNA modification and obesity-associated processes, and the underlying mechanism may involve the stabilization of a set of pseudogenes and poorly characterized predicted genes in mouse tissues, enabling their participation in the regulation of obesity (Pandey et al., 2020). Another investigation highlighted that m^6^Am-modified mRNAs are prominently enriched in various obesity- and metabolism-related processes in both lean and obese mice (Ben-Haim et al., 2021).

Furthermore, in obese mice subjected to a high-fat western diet (HFD), notable disruptions in the expression of FTO and its m^6^Am-modified targets were observed compared to chow diet-fed lean control mice (Ben-Haim et al., 2021). Specifically, several obesity-related genes, such as fatty acid binding protein 2 (FABP2) and FABP5, exhibited a loss of m^6^Am in their mRNAs, leading to a significant downregulation of protein expression due to the overexpression of FTO under HFD conditions (Ben-Haim et al., 2021). These findings provide compelling evidence supporting the involvement of m^6^Am RNA modification and its modifying enzymes in the regulation of metabolic pathways relevant to obesity.

### m^6^Am RNA modification and viral infection

Some viral RNAs undergo m^6^Am modification, and the process of viral infection is associated with m^6^Am RNA modification (Li and Rana, 2022). Recent discoveries have revealed the presence of cap m^6^Am in negative-sense RNA viruses, including vesicular stomatitis virus (VSV), rabies virus (RABV), and measles virus, which are generated by the host methyltransferase PCIF1 (Tartell et al., 2021). Deletion of *PCIF1* or expression of its catalytically inactive mutant augments the suppression of VSV and RABV gene expression by interferon-β treatment (Tartell et al., 2021).

In the case of human immunodeficiency virus (HIV), the causative agent of acquired immunodeficiency syndrome, its infection leads to the reduction of m^6^Am levels in ∼1/3 of modified genes within T cells (Zhang et al., 2021). Notably, in uninfected T cells, the enzyme PCIF1 plays a significant role by stabilizing ETS proto-oncogene 1 (*ETS1*) mRNA to promote its expression via methyltransferase activity (Zhang et al., 2021). This regulation enhances the function of ETS1 as a transcription factor to impede the transcription of HIV genomic DNA, serving as a protective mechanism against HIV infection (Zhang et al., 2021). However, the complexity of HIV–host interactions is evident, as HIV viral protein R (Vpr) interacts with PCIF1 to induce proteasome degradation, leading to a global reduction of m^6^Am in T cells, which potentially promotes viral replication (Zhang et al., 2021).

During the past global health crisis, severe acute respiratory syndrome coronavirus 2 (SARS-CoV-2) has garnered increasing attention as the causative agent. SARS-CoV-2 relies on the cell-surface proteins angiotensin converting enzyme 2 (ACE2) and transmembrane serine protease 2 (TMPRSS2) for entry into host cells (Jackson et al., 2022). A recent study demonstrated the pivotal role of PCIF1 in SARS-CoV-2 pathogenesis: it promotes the mRNA stabilization of *ACE2* and *TMPRSS2* in an m^6^Am-dependent manner, which increases the susceptibility of host cells to viral entry (Wang et al., 2023b). Collectively, these studies demonstrate the intricate and dual impacts of m^6^Am on different viral substrates, providing valuable insights into virus-associated pathogenesis and offering potential avenues for the development of targeted therapeutic strategies against virus-related diseases.

### m^6^Am RNA modification and cancers

Emerging studies have revealed that m^6^Am modification of RNAs plays critical roles in cancer development. Our group found that PCIF1 expression was significantly elevated in gastric cancer tissues, which was associated with poorer overall survival in patients (Zhuo et al., 2022). Mechanistically, the upregulation of PCIF1 expression enhances gastric cancer cell proliferation and invasion by increasing the m^6^Am level of *TM9SF1* mRNA to enhance its translation (Zhuo et al., 2022).

In colorectal cancer (CRC) patient-derived samples, FTO is found at low expression levels, whereas PCIF1 exhibits high expression levels (Relier et al., 2021; Wang et al., 2023a). Downregulation of FTO increases the abundance of m^6^Am and enhances the tumorigenic capability of CRC cells, enabling them to form tumor spheres and tolerate combined chemotherapy with 5-flurouracil and 7-ethyl-10-hydroxycamptothecin, while PCIF1 overexpression in CRC cells contributes to malignant behaviors, including increased cell proliferation, migration, invasion, fibronectin adhesion, and colony formation, indicating its engagement in multiple stages of tumorigenesis (Wang et al., 2023a). Moreover, *Pcif1* knockout in CRC tumors renders them sensitive to anti-PD-1 immunotherapy (Wang et al., 2023a). The function of m^6^Am in this regulatory axis is context dependent: it stabilizes the proto-oncogene *Fos* mRNA to promote tumor growth while destabilizing *STAT1* and *IFITM3* mRNAs to resist anti-PD-1 treatment (Wang et al., 2023a).

Intriguingly, PCIF1 has low expression levels in high-grade glioma tissues (Gao et al., 2022). Overexpression of PCIF1, but not its catalytically inactive mutant, results in the repression of glioma cell proliferation (Gao et al., 2022). These findings highlight that m^6^Am modification plays important roles in the regulation of cancer progression, with the underlying molecular mechanisms highly context dependent.

## Conclusion and perspectives

Despite the growing interest in m^6^Am RNA modification, several critical technical, biological, and clinical challenges remain to be addressed. From a technical standpoint, future advancements are expected to introduce more reliable methodologies, particularly those independent of antibodies. Technologies such as direct RNA-seq show promise, providing a comprehensive view of the RNA modification landscape without concerns related to antibody cross-reactivity and variations in binding affinity. Furthermore, third-generation sequencing platforms, characterized by longer read lengths and the ability to directly sequence RNA molecules, may enhance the accuracy of distinguishing between m^6^A and m^6^Am. From a biological perspective, the seemingly contradictory roles of m^6^Am modification in mRNA stability and translation, as well as its highly context-dependent functions during disease pathogenesis, need further investigation. Many aspects of m^6^Am are still largely unexplored, including the identification of m^6^Am ‘readers’ and their potential contribution to the diverse context-dependent roles of m^6^Am modification. Moreover, it is plausible that m^6^Am is involved in various diseases beyond obesity, viral infection, and cancers. For instance, studying whether mutations in m^6^Am-modifying enzymes lead to the loss of catalytic activity in disease contexts will provide valuable insights. Additionally, exploring the potential roles of m^6^Am in other physiological processes is essential for a comprehensive understanding of its functions. Regarding clinical applications, the systematic evaluation of m^6^Am levels in patient-derived materials, such as serum cell-free RNAs, shows promise in using m^6^Am as a novel biomarker for early disease diagnosis. Furthermore, there is a keen anticipation for the development of small-molecule inhibitors that target dysregulated m^6^Am-modifying enzymes and RNA editing tools that manipulate m^6^Am modification in mRNAs, which both have significant therapeutic implications.
